# Belatacept As an Alternative to Calcineurin Inhibitors in Patients with Solid Organ Transplants

**DOI:** 10.3389/fmed.2017.00060

**Published:** 2017-05-19

**Authors:** Dhiren Kumar, Spencer LeCorchick, Gaurav Gupta

**Affiliations:** ^1^Division of Nephrology, Virginia Commonwealth University, Richmond, VA, USA; ^2^Division of Transplant Surgery, Virginia Commonwealth University, Richmond, VA, USA

**Keywords:** kidney transplantation, solid organ transplantation, T cell costimulation, immunosuppression, graft rejection

## Abstract

The goal of immunosuppression in transplantation has shifted to improving long-term outcomes, reducing drug-induced toxicities while preserving the already excellent short-term outcomes. Long-term gains in solid organ transplantation have been limited at least partly due to the nephrotoxicity and metabolic side effects of calcineurin inhibitors (CNIs). The alloimmune response requires activation of the costimulatory pathway for T cell proliferation and amplification. Belatacept is a molecule that selectively blocks T cell costimulation. In June 2011, the U.S. Food and Drug Administration approved it for maintenance immunosuppression in kidney transplantation based on two open-label, randomized, phase III trials. Since its introduction, belatacept has shown promise in both short- and long-term renal transplant outcomes in several other trials. It exhibits a superior side effect profile compared to CNIs with a comparable efficacy. Across all solid organ transplants, the burden of chronic kidney disease, its associated cardiovascular morbidity, mortality, and inferior patient/allograft survival is a well-documented problem. In this review, we aim to discuss the evidence behind the use of belatacept in solid organ transplants as an effective alternative to CNIs for renal rescue in patients with acute and/or chronic kidney injury.

## Introduction

Transplantation is the treatment of choice for end stage organ failure. In non-renal solid organ transplants, with no replacement therapies, transplantation is a lifesaving procedure. When compared to dialysis, kidney transplantation provides patients with a better quality of life and decreased cardiovascular morbidity and mortality (1). The introduction of calcineurin inhibitors (CNIs) has reduced acute rejection rates and markedly improved short-term graft survival (1 year-93%). A commensurate improvement in long-term graft (5 years-72%, 10 years-46%) and patient survival still leaves much to be desired (2–4).

Chronic renal failure is a common complication of non-renal solid organ transplantation. The presence of chronic renal failure is associated with an increased risk of death (relative risk, 4.55; *p* < 0.001) (5, 6). At 3 years posttransplant, chronic renal failure developed in 16.5% of patients of whom 29% required either dialysis or renal transplantation. The risk of renal failure increases over time in patients with non-renal solid organ transplants (7). There is substantial evidence to suggest that CNIs lead to nephrotoxicity and contribute to progression of chronic kidney disease in this patient population (8).

In renal transplantation, the leading causes of death and graft loss are cardiovascular disease and chronic allograft nephropathy (9). These drugs that are the mainstay of modern day immunosuppression contribute to cardiovascular mortality due to their deleterious effects on blood pressure, dyslipidemias, and diabetes (10). Successful renal transplantation may increase life expectancy by 10–20 years; however, the annual risk of death by cardiovascular causes is 50-fold higher in kidney transplant recipients when compared to the general population (11).

Graft survival in the long run is impaired by the nephrotoxicity of CNIs. Five years after transplantation histological evidence of CNI induced nephrotoxicity is seen in greater than 50% of allografts, and by 10 years, this finding is universal (12).

Many alternative regimens such as CNI minimization, withdrawal and avoidance with use of mammalian target of rapamycin (mTOR) inhibitors (sirolimus and everolimus) have been tried but rejection risks are higher, and sustained improvement in kidney function has not been seen consistently across different studies (13–15).

The overall goal of immunosuppression in transplantation includes minimization of acute and chronic rejection while managing the adverse cardiovascular, nephrotoxic, infectious, and malignant sequelae of chronic immunosuppression.

Belatacept (Bristol-Myers Squibb, New York, NY, USA), a molecule that blocks T cell costimulation, has shown promise in both short- and long-term renal transplant outcomes with a superior side-effect profile compared to CNIs (16).

## Aims and Methods

In this review, we aim to discuss the mechanism of action of belatacept, its role in kidney transplantation as an effective alternative to CNIs, and its future in non-kidney solid organ transplantation.

### Mechanism of Action and Preclinical Trials of Belatacept

Many immunosuppressive agents in use today interfere with specific steps that lead to T cell activation. It was theorized that a CTLA4-Ig protein with high affinity for CD80/86 could attenuate the T cell response by blocking “Signal 2” or costimulation. Abatacept was the first such molecule that was developed. Even though it proved successful in treating diseases mediated by an autoimmune T cell response (rheumatoid arthritis and psoriasis) it had an inadequate response in non-human primate transplant models (17–19).

Using a rational mutagenesis and screening strategy, a daughter molecule LEA29Y (belatacept) with two amino acid substitutions (L104 → E and A29 → Y) was developed (20). Belatacept was found to bind four times more avidly to CD86 and two times more avidly to CD80 than the parent abatacept. This improved binding resulted in approximately 10-fold more potent inhibition of T cell activation (20).

Preclinical trials in non-human primate transplant models by Kirk et al. and Montgomery et al. revealed that treatment with anti-CD80 and anti-CD86 monoclonal antibodies delayed allograft rejection but did not induce tolerance (21, 22). The pioneering study by Larsen et al. showed that LEA29Y in combination with conventional immunosuppression was synergistic and superior with respect to graft survival when compared to LEA29Y alone (20).

### *De Novo* Belatacept in Renal Transplantation

#### Belatacept Evaluation of Nephroprotection and Efficacy As First-line Immunosuppression Trial (BENEFIT) and Belatacept Evaluation of Nephroprotection and Efficacy As First-line Immunosuppression Trial—EXTended Criteria Donors (BENEFIT-EXT) Studies: Short-term Outcomes

The use of belatacept as first-line immunosuppression was first published in a phase III study by Vincenti et al. (BENEFIT study) (9). This study randomized patients to three groups: cyclosporine (CsA) (*n* = 221), less intensive (LI) belatacept (*n* = 226), and more intensive (MI) belatacept (*n* = 219). Induction regimen included basiliximab and maintenance immunosuppression included mycophenolate mofetil (MMF) and corticosteroids. At 12 months, the mean measured estimated glomerular filtration rate (eGFR) was higher in the belatacept arms as compared to the CsA group by 13–15 ml/min. Episodes of acute rejection at 12 months were higher in the belatacept groups compared with CsA (22% MI; 17% LI; 7% CsA). The majority of acute rejection episodes occurred in the first 3 months, and all of the rejection occurred within 6 months of transplantation. Despite higher rejection rates in the belatacept groups, the mean measured GFR at month 12 was higher than in the CsA group without acute rejection. No cases of antibody-mediated rejection were reported, and development of *de novo* anti-HLA donor-specific antibody (DSA) was lower in the belatacept groups (3% MI; 1% LI; 7% CsA). The prevalence of biopsy-proven chronic allograft nephropathy at month 12 was lower in the belatacept groups compared with CsA (18% MI; 24% LI; 32% CsA). There was no statistical difference in patient and graft survival at the end of 1 year between all three groups (95% MI; 97% LI; 93% CsA).

The belatacept treated group had better cardiovascular and metabolic outcomes. Mean blood pressure (systolic and diastolic) were statistically lower in patients treated with either belatacept regimen when compared to the CsA group. Mean systolic blood pressure was 133, 131, and 139 mmHg in the belatacept MI, belatacept LI, and CsA group, respectively (*p* = 0.001 MI vs CSA, *p* < 0.0001 LI vs CsA). Mean non-HDL cholesterol increased by 8.1, 8.0, and 18.3 mg/dl in the belatacept MI, belatacept LI, and CsA arms, respectively (*p* = 0.01 MI vs CSA, *p* = 0.01 LI vs CsA). Patients in either belatacept arm had a significant reduction in triglycerides; −23.6 mg/dl (belatacept MI, *p* = 0.0165 vs CsA), −27.8 mg/dl (belatacept LI, *p* = 0.0047 vs CsA) compared to an increase in the CsA group, +6.6 mg/dl. There was no statistically significant difference in the incidence of new onset diabetes after transplant (NODAT) between the belatacept treatment arms and the CsA treatment arm.

An extended criteria donor (ECD) is any donor who at the time of death is over the age of 60, or over the age of 50 with two of the following three criteria: (1) history of high blood pressure, (2) a terminal creatinine of >1.5 mg/dl, and (3) death by cerebrovascular accident. ECD kidney allografts are associated with delayed graft function (DGF) and poorer long-term outcomes (23). In recipients of ECD kidneys, donors deceased after cardiac death and elderly donors with preexisting vascular lesions, arteriolar vasoconstriction could lead to acute allograft dysfunction, which could promote chronic interstitial fibrosis and tubular atrophy (24).

*De novo* use of belatacept in ECD kidneys was studied in the BENEFIT-EXT study. The mean measured GFR at 12 months was significantly different in the MI belatacept (52.1 ml/min/1.73 m^2^) vs the CsA group (45.2 ml/min/1.73 m^2^) *p* = 0.008. There was no difference between the belatacept LI group (49.5 ml/min/1.73 m^2^) and CsA group, *p* = 0.1. Similar to the BENEFIT study, the belatacept-treated groups had non-inferior patient and graft survival compared to the CsA group (86% MI; 89% LI; 85% CsA). The rates of DGF were also similar between the groups (47% MR; 47% LI; 49% CsA).

The belatacept treated groups in the BENEFIT-EXT study also had better cardiovascular and metabolic outcomes. Mean non-HDL cholesterol increased by 12.6, 11.2, and 29.3 mg/dl in the belatacept MI, belatacept LI, and CsA arms, respectively (*p* = 0.0016 MI vs CSA, *p* = 0.0006 LI vs CsA). Mean triglycerides decreased by 1 mg/dl (belatacept MI) and 18.2 mg/dl (belatacept LI) compared to an increase of 34.5 mg/dl in the CsA arm (*p* = 0.0106 MI vs CSA, *p* = 0.0001 LI vs CsA). Incidence of NODAT at 12 months was lower in the belatacept MI group (2%) compared to the CsA group 5% (*p* = 0.03 MI vs CsA) (25).

#### BENEFIT and BENEFIT-EXT Study: Long-term Outcomes

The 7-year outcomes from the BENEFIT study have yielded encouraging results. One hundred fifty-three of the 219 patients (70%) treated with the MI belatacept regimen; 163 of the 226 (72%) treated with the LI belatacept regimen; and 131 of the 215 (61%) treated with CsA were followed for the full 7-year period. While the results were not conclusive (likely due to inadequate power), there was a trend toward a decrease in the likelihood of death at 7 years by 38% (HR 0.62, 95% CI, 0.33–1.14; *p* = 0.11) in the MI belatacept group and by 45% (HR 0.55, 95% CI, 0.30–1.04; *p* = 0.06) in the LI belatacept group when compared with the CsA group. Similarly, the likelihood of graft loss at 7 years was reduced by 44% (HR 0.56, 95% CI, 0.26–1.23; *p* = 0.12) in the MI belatacept group and by 41% (0.49, 95% CI, 0.22–1.09; *p* = 0.07) in the LI belatacept group.

Over the 7 years, the belatacept groups had a sustained yearly improvement in eGFR while the CsA group had a persistent decline in eGFR. At 12, 36, 60, and 84 months, the mean eGFR values were 67.0, 68.9, 70.2, and 70.4 ml/min/1.73 m^2^ of body-surface area, respectively, with MI belatacept and 66.0, 68.9, 70.3, and 72.1 ml/min/1.73 m^2^ with LI belatacept. This was much improved when compared to the corresponding declining values for CsA: 52.5, 48.6, 46.8, and 44.9 ml/min/1.73 m^2^.

Development of *de novo* DSA remained markedly lower in the belatacept groups when compared with the CsA group. Rates of development of donor-specific antibodies at months 36, 60, and 84 were 1.2, 1.9, and 1.9%, respectively, with the MI belatacept regimen and 3.4, 4.6, and 4.6% with the LI belatacept regimen. The corresponding values for CsA were 8.7, 16.2, and 17.8% (16).

Seven-year outcomes from the BENEFIT-EXT study were also reported recently. Of the original 543 patients who comprised the intent-to-treat population, 372 (69%) had data available for analysis at 7 years; 128 of the 184 (70%) treated with the MI belatacept regimen, 138 of the 175 (79%) treated with the LI belatacept regimen, and 108 of the 184 (59%) treated with CsA. At 7 years, there was no difference in the likelihood of death between the belatacept MI (HR 1.108; 95% CI 0.679–1.808; *p* = 0.69) or belatacept LI group (HR 1.119; 95% CI 0.688–1.822; *p* = 0.66) and the CsA group. Similarly, there was no significant difference in death censored graft lost at 7 years between the belatacept MI regimen (12.4%; HR 0.700; 95% CI 0.399–1.228; *p* = 0.21), or belatacept LI regimen (13.6%; HR 0.783; 95% CI 0.452–1.353; *p* = 0.36) and the CsA group (19.3%).

Over the 7 years, the ECD LI belatacept treated group showed a sustained yearly improvement in eGFR while the CsA treated group had a persistent decline in eGFR. The MI belatacept group showed an overall improvement in eGFR. At 12, 36, 60, and 84 months, the mean eGFR values were 48.3, 52.5, 52.2, and 53.9 ml/min/1.73 m^2^ of body-surface area, respectively, with MI belatacept and 47.8, 50.1, 52.7, and 54.2 ml/min/1.73 m^2^ with LI belatacept. This was improved when compared to the corresponding declining values for CsA: 40.3, 38.0, 35.8, and 35.3 ml/min/1.73 m^2^.

Development of *de novo* DSA remained lower in the belatacept groups when compared to the CsA treated group. Rates of development of donor-specific antibodies at months 36, 60, and 84 were 2.3, 6.2, and 6.2%, respectively, with the MI belatacept regimen and 1.5, 2.4, and 4.5% with the LI belatacept regimen. The corresponding values for CsA were 11.3, 17.1, and 22.9% (26). Data from the BENEFIT and BENEFIT-EXT studies are summarized in Table 1.

**Table 1:** **Clinical trials of *de novo* belatacept in renal transplantation**.

Trial	Treatment groups	Acute rejection	Graft loss	GFR at end of study (ml/min/1.73 m^2^)	Notes
Phase III, randomized, partially blinded, multicenter (BENEFIT)	MI Belatacept	49/219 (22%)	4/219 (2%)	65^a^	Basiliximab induction with MMF + steroids as maintenance

	LI Belatacept	39/226 (17%)	5/226 (2%)	63^a^	

1-year outcomes (9)	CsA	16/221 (7%)	8/221 (4%)	50	

Phase III, randomized, partially blinded, multicenter (BENEFIT)	MI Belatacept	54/219 (24%)	25/219 (12.7%)	70^b^	Basiliximab induction with MMF + steroids as maintenance

	LI Belatacept	41/226 (18%)	26/226 (12.8%)	72^b^	

7-year outcomes (16)	CsA	24/221 (11%)	40/221 (21.7%)	45	

Phase III, randomized, partially blinded, multicenter (BENEFIT-EXT)	MI Belatacept	33/184 (18%)	17/184 (9%)	52^a^	Basiliximab induction with MMF + steroids as maintenance

	LI Belatacept	31/175 (18%)	16/175 (9%)	49^c^	

1-year outcomes (25)	CsA	26/184 (14%)	20/184 (11%)	45	

Phase III, randomized, partially blinded, multicenter (BENEFIT-EXT)	MI Belatacept	39/184 (21.1%)	21/184 (12.4%)	53.9^b^	Basiliximab induction with MMF + steroids as maintenance

	LI Belatacept	34/175 (19.5%)	23/175 (13.6%)	54.2^b^	

7-year outcomes (26)	CsA	32/184 (17.3%)	29/184 (19.3%)	35.3	

*^a^p < 0.01 vs standard CsA*.

*^b^p < 0.001 vs standard CsA*.

*^c^p = 0.1 vs standard CsA*.

*MI Belatacept, more intensive belatacept; LI Belatacept, less intensive belatacept; CsA, cyclosporine A; CNI, calcineurin inhibitor; MMF, mycophenolate mofetil; GFR, glomerular filtration rate; BENEFIT, Belatacept Evaluation of Nephroprotection and Efficacy as First-line Immunosuppression Trial; BENEFIT-EXT, Belatacept Evaluation of Nephroprotection and Efficacy as First-line Immunosuppression Trial—EXTended criteria donors*.

Cardiovascular outcomes were not reported in the 7-year outcomes from the BENEFIT or BENEFIT-EXT trial. Cardiovascular outcomes appeared to be improved at 1 year. In both trials at 7 years, patients randomized to the two belatacept treatment arms had improved renal function but failed to show improved patient or graft survival. Longer follow-up in needed to determine the long-term benefits of a belatacept-based/CNI-free immunosuppression regimen.

Ferguson et al. reported 1-year data from an open-labeled phase II trial evaluating immunosuppression with a belatacept-based corticosteroid avoidance regimen in *de novo* kidney transplant recipients. Patients were randomized in a 1:1:1 ratio to either belatacept–MMF (*n* = 33), belatacept–sirolimus (*n* = 26), or Tac–MMF (*n* = 30). All patients received induction with thymoglobulin, 6 mg/kg total dose. At 6 months, four patients in the belatacept–MMF group (12%), one in the belatacept–sirolimus (4%), and one in the Tac–MMF (3%) had acute rejection. At 12 months, one additional patient in the belatacept–MMF group had acute rejection. Patient survival with a functioning graft was similar at 12 months; 91, 92, and 100% in the belatacept–MMF, belatacept–sirolimus, and Tac–MMF groups, respectively. Renal function was better in both belatacept groups compared to the Tac–MMF group. Mean calculated GFR was 63.6 ml/min/1.73 m^2^ in the belatacept–MMF group, 61.8 ml/min/1.73 m^2^ in the belatacept–sirolimus group, and 54.0 ml/min/1.73 m^2^ in the Tac–MMF group. No difference in cardiovascular risk factors including blood pressure, cholesterol, or new onset diabetes was noted (27). This trial provides initial data about the efficacy of a steroid free belatacept-based immunosuppression in combination with anti-T cell depleting induction therapy. Major limitations include small sample size, inadequate power, and limited follow-up. More robust phase III trials are need.

A Cochrane database review published in 2016 by Masson et al. compared belatacept- and CNI-based immunosuppression regimens. The findings mirrored and summarized the findings in the BENEFIT and BENEFIT-EXT studies. Overall, the use of belatacept was associated with better graft function (eGFR: mean difference 9.96 ml/min/1.73 m^2^, 95% CI 3.28–16.64) and less risk of chronic kidney scarring (RR 0.72, 95% CI 0.55–0.94) as compared to the CNI-treated transplant recipients. There was no statistically significant difference in risk of death, loss of allograft, and acute rejection. Cardiovascular and metabolic outcomes also were congruent with the BENEFIT study with lower blood pressures, better lipid profiles, and reduced incidence of NODAT (RR 0.61, 95% CI 0.40–0.93) in the belatacept-treated groups (28).

There are major limitations in the BENEFIT and BENEFIT-EXT studies that make it difficult to apply these data to a wide patient population. Patients in both studies received basiliximab induction therapy. In the latest OPTN/SRTR Annual Data Report, it was reported that over 60% of kidney transplant patients received induction immunosuppression with a T-cell depleting agent compared to only 33% of patient who received an IL-2 receptor antagonist. In addition, tacrolimus has replaced CsA as the CNI of choice (29). Overall, the patient population in these trials were immunologically low risk. In the BENEFIT study, patients were included if the cold ischemia time was <24 h. Patients were excluded if they had a previous non-renal transplant, simultaneous organ transplant, panel reactive antibody (PRA) >50% in first-time renal transplant patients, and PRA >30 in regrafts (9). Twelve month biopsy results were not available for all enrolled patients. Finally, there was evidence of increased non-adherence in the CsA groups compared with the belatacept groups. This could have biased the results in favor of the belatacept groups.

### Conversion to Belatacept in Renal Transplantation

To avoid prolonged exposure to CNIs, Rostaing et al. in a phase II study randomized kidney transplant recipients to switch from a CNI-based regimen to a belatacept-based regimen (Table 2). Patients who were 6 months posttransplant with stable graft function were randomized to belatacept conversion (*n* = 84) or remained on their CNI-based regimen (*n* = 89). At 1 year, the mean GFR improved by 7 ml/min/1.73 m^2^ in the belatacept group as compared to 2.1 ml/min/1.73 m^2^ in the CNI group (*p* = 0.0058). There were six (7%) cases of acute rejection in the belatacept group as compared to none in the CNI group. No grafts were lost in the belatacept group, and one patient died with a functioning graft in the CNI group due to myocardial infarction. Cardiovascular and metabolic outcomes did not differ clinically between the two groups (30).

**Table 2:** **Clinical trials of belatacept conversion in renal transplantation**.

Trial	Treatment groups	Acute rejection	Graft loss	GFR change (ml/min/1.73 m^2^) per year	Notes
Phase II, randomized	Belatacept	6/84 (7%)	0/84 (0%)	+7^a^	1-year results, patients converted after 6 months with stable renal function
	
Switching CNI to belatacept (30)	CsA/Tac	0/89 (0%)	1/89 (1.1%)	+2	

Phase II, randomized	Belatacept	6/84 (7%)	1/84 (1.2%)	+9	2-year results, patients converted after 6 months with stable renal function
	
Switching CNI to belatacept 2-year outcomes (31)	CsA/Tac	3/89 (3%)	2/89 (2.2%)	+0.3	

Phase II, randomized	Belatacept	7/84 (8.4%)	2/84 (2.4%)	+1.9^b^	3-year results, patients converted after 6 months with stable renal function 3 year outcomes (32)
	
Switching CNI to belatacept	CsA/Tac	3/89 (3.6%)	2/89 (2.2%)	+0.07	

Retrospective review					
Early switch from CNI to belatacept in extended criteria donor kidney donor transplantation (33)	CNI converted to Belatacept	1/25 (4%)	11/25 (44%)	+17^c^	Patients converted for prolonged delayed graft function and/or poor allograft function

*^a^p = 0.0058 for difference between treatment groups in change from baseline*.

*^b^p = 0.01 for difference between treatment groups in change from baseline*.

*^c^p = 0.001*.

*CsA, cyclosporine A; CNI, calcineurin inhibitor; MMF, mycophenolate mofetil; GFR, glomerular filtration rate; Tac, tacrolimus*.

Two-year results from the same study population continued to show a sustained improvement in GFR in the belatacept group (Table 2). From baseline, the mean GFR improved by 8.8 ml/min/1.73 m^2^ in the belatacept group as compared to 0.3 ml/min/1.73 m^2^ in the CNI group. The mean difference in GFR between the two groups rose from 4.3 ml/min/1.73 m^2^ to 8.5 ml/min/1.73 m^2^ in favor of the belatacept group. The higher rate of rejection seen in the first year post-conversion did not persist in the second year. There were no additional episodes of rejection seen from year 1 to 2 in the belatacept group. Patient and graft survival at the end of 2 years were equivalent in both groups (31).

Three-year results from the same group showed continued improvement in eGFR. At three years, eGFR increased by 1.9 ml/min/1.73 m^2^ per year in the group switch to a belatacept-based immunosuppression regimen compared to 0.07 ml/min/1.73 m^2^ in the group who remained on a CNI-based regimen (*p* = 0.01). At 3 years 8.4% of patients converted to belatacept had experienced an acute rejection compared to 3.6% of patients who remained on a CNI (HR 2.50 95% CI, 0.65–9.65; *p* = 0.2). There was no difference in patient or graft survival between groups. A trend in improvement of renal function may have been blunted by the addition of 16 patients who were switched from a CNI-based immunosuppression regimen to belatacept after 24 months post-randomization (32).

Conversion to belatacept in patients with allograft dysfunction after exposure and established intolerance to CNI-based regimens has also revealed encouraging results. In a retrospective analysis, Le Meur et al. looked at 25 patients who were converted to belatacept for indications of prolonged DGF and/or poor allograft function. All but one patient in this study received ECD kidneys. Twenty-four of the 25 patients (96%) underwent pre-conversion biopsies. The majority of these biopsies revealed histological patterns of acute tubular injury due to ischemia reperfusion and/or donor-derived vascular lesions, which are associated with renal dysfunction and CNI nephrotoxicity. Having failed CNI minimization, the patients were converted to belatacept-based regimen within 6 months of transplantation. Six months after conversion 22 (88%), at 12 months 20 (80%), and at 24 months 14 (56%), patients had functioning grafts. Three patients did not recover kidney function. Of the remaining 22 patients, renal function improved from a nadir eGFR of 18.28 ± 12.3 ml/min before conversion to a sustained long-term improvement of 33.9 ± 10.1 ml/min at 6 months (*p* < 0.001), 34.9 ± 14.5 ml/min at 1 year (*p* = 0.001), and 35.6 ± 13.6 ml/min at 2 years (*p* = 0.001) (Table 2). One patient suffered acute cellular rejection that was reversed completely with methylprednisolone pulse treatment and remained on belatacept (33).

Data from two German transplant centers were recently published detailing 79 kidney transplant patients with chronic renal allograft dysfunction who were converted to belatacept from a CNI or mTOR inhibitor. The mean time from transplant to belatacept conversion was 69 months. The major indication for conversion was biopsy confirmed CNI toxicity (51.9%). Seventeen of the 22 patients who were switched from an mTOR inhibitor had previously been switched from a CNI due to biopsy confirmed CNI toxicity. Mean eGFR at the time of conversion was 26.1 ± 15 ml/min/1.73 m^2^ and increased to 29.7 ± 15.0 ml/min/1.73 m^2^ (*p* = 0.002), 31.6 ± 14.5 ml/min/1.73 m^2^ (*p* < 0.005), and 34.0 ± 15.2 ml/min/1.73 m^2^ (*p* < 0.005) at 3, 6, and 12 months post-conversion, respectively. Proteinuria improved at all time points’ post-conversion but did not differ significantly from baseline. Nine (11.4%) patients experienced biopsy-proven acute rejection; of these two suffered graft loss shortly after conversion to belatacept (34).

Based on data from these trials conversion from CNI-based immunosuppression regimen to a belatacept-based regimen appears safe with a slight increase in acute rejection soon after conversion. Patients converted to belatacept have been shown to have stabilization of renal function and some experience improvement in eGFR. Long-term data are need to assess whether short-term improvements in renal function lead to improvements in graft survival.

### Belatacept Conversion for High-Immunologic Risk Patients

Highly sensitized recipients are at a disadvantage due to the small available donor pool. This results in pressure to use marginal donor kidneys. This combination could come with unfavorable donor-derived histological characteristics and along with chronic alloimmune mediated injury could predispose the recipient to early allograft dysfunction and poor long-term outcomes. Even in a low immunological risk population, higher rates of acute rejection were seen in the BENEFIT study. This led to a natural aversion to using belatacept in this group of patients. There is almost no literature on the use of belatacept in the sensitized patient population. In our initial experience reported in the American Journal of Transplantation, we converted six highly sensitized patients with a median calculated panel reactive antibody of 88% to a belatacept-based regimen. Indications for conversion were biopsy-proven CNI toxicity and/or interstitial fibrosis and tubular atrophy along with poor allograft function. MMF was maximized to a dose of 2–3 g/day as tolerated, and tacrolimus was weaned off at a slower rate than published previously over a period of 2–3 months. Renal function improved significantly from a mean eGFR of 23.8 ± 12.9 ml/min/1.73 m^2^ prior to the conversion to an eGFR of 42 ± 12.5 ml/min/1.73 m^2^ (*p* = 0.028) at the end of a mean follow-up of 16.5 months. Surveillance biopsies after conversion were performed on five of six patients. Histologically, no new episodes of rejection were observed, and there was no worsening of chronicity scores. Immunologically, there was no emergence of new DSA. One patient who had DSA prior to conversion had declining DSA post-conversion without histological evidence of rejection (35).

Data are lacking to recommend conversion to belatacept from a CNI-based immunosuppression regimen in highly sensitized patients. Additional follow-up to assess new DSA formation, rates of antibody-mediated rejection, and renal function is needed.

### Belatacept and Thrombotic Microangiopathy (TMA)

The incidence of *de novo* TMA posttransplant has been reported as 0.8–3.3% (36, 37). Drug-induced TMA has been associated with both CNIs (38, 39) and with mTOR inhibitors (40). Conventional therapies such as withdrawal of the offending drug (usually CNI), switching drugs, and plasma exchange have resulted in poor graft outcomes. Drug withdrawal has been associated with a 50% graft survival rate at 3 years (36). Plasma exchange has been associated with an immediate graft loss rate of 20% and 1-year graft survival of only 66% (41). There continues to be no treatment guidelines of *de novo* TMA recurrence posttransplant.

Successful conversion to belatacept in the setting of drug-induced TMA has been described in case series (42) and case reports (43, 44). These reports have demonstrated encouraging short-term outcomes after withdrawal of CNI and/or sirolimus.

### Belatacept in Non-Kidney Solid Organ Transplantation

#### Liver Transplantation

A phase II randomized multicenter trial was the first to explore the *de novo* use of belatacept in liver transplant recipients. The study by Klintmalm et al. compared the 1-year outcomes of a belatacept- vs tacrolimus-based maintenance immunosuppression in liver transplantation. At the conclusion of this study, two of three belatacept groups had higher rates of death and graft loss (42–48%) relative to the standard-of-care tacrolimus and MMF (15%) control group (45). The belatacept arms had a higher incidence of death primarily related to infections. The etiology behind this increased risk of infections remains unclear. Liver transplant patients are more immunocompromised at baseline than kidney transplant patients, and perhaps the CD28 pathway is critical to their protective immunity (46). At 12 months, all three belatacept groups also had higher rates of acute rejection (31–40%) as compared with the control group (13%). The glomerular filtration rate (GFR) was better in the belatacept group as compared with the tacrolimus group. A total of 47–65% of patients had a greater than 10 ml/min improvement in GFR at 12 months vs 12–27% in the tacrolimus groups. However, these findings did not allow for the identification of a safe and effective role for belatacept in liver transplant recipients (45).

It is possible that the use of induction immunosuppression along with belatacept or short-term belatacept therapy might allow for preservation of the renal benefits of belatacept without compromising graft outcomes. In an interesting case series by LaMattina et al., the authors converted seven hepatitis C-positive liver transplant recipients to belatacept between 2 and 89 days posttransplant for renal dysfunction. All patients had a creatinine clearance of less than 35 ml/min/1.73 m^2^ at the time of conversion, and three of them were on dialysis. All patients received basiliximab-based induction. After conversion to belatacept, all patients recovered renal function to a point that they could be transitioned back to CNIs. The mean calculated GFR improved to 86 ± 23 ml/min/1.73 m^2^. One biopsy-proven rejection episode was observed while on belatacept. This patient had already suffered acute rejection while being maintained on low dose CNI therapy to preserve renal function. All patients and grafts survived the duration of belatacept therapy. Six month graft and patient survival was 86% (47).

With the available data the use of belatacept cannot be recommended in liver transplant recipients. Additional studies with sufficient power are needed to determine efficacy of belatacept in the liver transplant population.

#### Lung Transplantation

In a retrospective analysis by Timofte et al., eight lung transplant recipients with renal dysfunction had belatacept added to their regimen with further reduction or complete discontinuation of CNIs. These patients had failed or were deemed unsuitable for isolated CNI minimization and mTOR-based avoidance protocols. They were then compared with a historical cohort of lung transplant recipients who had renal dysfunction and did not receive belatacept. There was a trend toward improved renal function in the belatacept treated group. One episode of low-grade steroid responsive acute rejection was seen in the belatacept group (48).

#### Heart Transplantation

The use of belatacept in heart transplantation has been reported in only one case report to date. Enderby et al. added belatacept to the immunosuppressive regimen of a 26–year-old recipient of an orthotopic heart transplant. This was done in an effort to improve suspected non-compliance after multiple episodes of rejection related to erratic tacrolimus and sirolimus levels. While on belatacept, surveillance biopsies revealed no new episodes of rejection; coronary angiogram revealed normal coronaries and echocardiography revealed a left ventricular ejection fraction of 61%. The patient died from cardiac arrest 6 months after addition of belatacept due to unclear reasons (49).

### Belatacept Safety and Adverse Events

The 7-year outcomes from the BENEFIT study reveal that belatacept has acceptable and comparable safety to traditional immunosuppressive regimens. The adverse event rates (69–71 vs 76%), serious infection rates (11 vs 13 events per 100 person-years of treatment), and cancer incidence rates (1.8–2.1 vs 2.6 events per 100 person-years of treatment) were equivalent to the CNI-based immunosuppression (16). Also, in the Cochrane database review, there was no difference in risk of any malignancy or infection in both treatment arms (28).

There was a higher incidence of posttransplant lymphoproliferative disease (PTLD) with belatacept in the first year after transplantation. Epstein–Barr virus (EBV) seronegative status was the strongest associated risk factor and represented a ninefold increase in risk (50). Owing to these concerns, current belatacept trial protocols have now been modified to enroll EBV seropositive patients only (51). The Food and Drug Administration has also provided a black box warning against using belatacept in EBV-seronegative patients. In the seven-year follow-up, only one case of PTLD was noted to occur after the first 24 months of therapy in the belatacept group (16).

## Discussion

Progressive chronic kidney disease is a well-known complication of all solid organ transplants. Prior disease, peri-operative insults, and long-term exposure of vasoconstrictive CNIs can all contribute to this. In addition, chronic kidney disease worsens cardiovascular morbidity and mortality and has been shown to be an independent risk factor for adverse transplant outcomes.

In kidney transplantation, the BENEFIT studies that investigated the use of *de novo* belatacept as compared to CsA showed that belatacept was associated with higher rates of early low-grade steroid responsive acute rejection. This was offset by higher GFR, lower blood pressures, and lower incidence of NODAT. A few important limitations of these studies included the use of CsA as the comparator of choice rather than the more potent tacrolimus. In addition, only low immunologic risk patients were enrolled. Long-term outcomes have now been reported to show a clear trend toward improved patient and graft survival. Nevertheless, it remains to be seen whether the improved metabolic profile leads to a reduction in cardiovascular morbidity and mortality.

From an immunological standpoint, the observed reduced rate of development of donor-specific antibodies in belatacept-treated groups has encouraging long-term implications. Inadequate immunosuppression either due to non-compliance or reduction in immunosuppression is the major cause of late DSA formation and chronic antibody-mediated rejection (52). Development of DSA and chronic antibody-mediated rejection has been implicated as a major cause of long-term graft loss (53–55). The nature of administration of belatacept that involves delivery by a trained health-care professional and once a month dosing might ensure better compliance and lesser variability in baseline immunosuppression. There is also some suggestion that belatacept might also diminish the B-cell responses (56).

In randomized conversion trials, as with the *de novo* studies, a higher rate of early acute rejection post-belatacept conversion was reported. Despite this, belatacept was shown to improve or stabilize intermediate-term kidney allograft function at up till 3 years follow-up. Thus, based on the data available so far, a careful conversion strategy might be safe in allografts with stable function and even those with dysfunction and established intolerance to CNIs. The findings of belatacept conversion studies that are ongoing are eagerly awaited.

The use of belatacept should be carefully individualized in kidney transplant recipients. Factors to be taken into account are the cardiovascular and hemodynamic complications associated with poor allograft function along with the immunological risk of the recipient. Future studies are underway to assess the safety and efficacy of belatacept in the setting of immunological sensitization and in combination with lymphocyte depleting agents. The results of these studies may help broaden the scope of this well-tolerated drug and make CNI free immunosuppression a real possibility for the vast majority of kidney transplant patients.

As outlined in this review, the non-kidney solid organ transplant experience with belatacept is sorely lacking. The role of belatacept is not yet established in this group of transplant patients. The scarce data that are available currently do not lend itself to generalizability. More studies are urgently needed in these end organ failure transplant groups where renal dysfunction remains a widely prevalent and important contributor to patient mortality.

## Author Contributions

DK, GG, and SL made substantial contributions to acquisition of data, and/or analysis and interpretation of data; participated in drafting the article or revising it critically for important intellectual content; gave final approval of the version to be submitted and any revised version; and agreed to be accountable for all aspects of the work in ensuring that questions related to the accuracy or integrity of any part of the work are appropriately investigated and resolved.

## Conflict of Interest Statement

GG has served on the Scientific Advisory Board of Bristol-Myers Squibb. The remaining authors declare that the research was conducted in the absence of any commercial or financial relationships that could be construed as a potential conflict of interest.

## References

[1] Knoll G. Trends in kidney transplantation over the past decade. Drugs (2008) 68(Suppl 1):3–10. 10.2165/00003495-200868001-00002 18442296

[2] Emamaullee J Toso C Merani S Shapiro AM. Costimulatory blockade with belatacept in clinical and experimental transplantation – a review. Expert Opin Biol Ther (2009) 9(6):789–96. 10.1517/14712590902942284 19426116

[3] Cooper JE Wiseman AC. Novel immunosuppressive agents in kidney transplantation. Clin Nephrol (2010) 73(5):333–43. 10.5414/CNP73333 20420793

[4] Matas AJ Smith JM Skeans MA Thompson B Gustafson SK Schnitzler MA OPTN/SRTR 2012 annual data report: kidney. Am J Transplant (2014) 14(Suppl 1):11–44. 10.1111/ajt.12579 24373166

[5] Wolfe RA Ashby VB Milford EL Ojo AO Ettenger RE Agodoa LY Comparison of mortality in all patients on dialysis, patients on dialysis awaiting transplantation, and recipients of a first cadaveric transplant. N Engl J Med (1999) 341(23):1725–30. 10.1056/NEJM199912023412303 10580071

[6] Mauger EA Wolfe RA Port FK. Transient effects in the Cox proportional hazards regression model. Stat Med (1995) 14(14):1553–65. 10.1002/sim.4780141406 7481192

[7] Ojo AO Held PJ Port FK Wolfe RA Leichtman AB Young EW Chronic renal failure after transplantation of a nonrenal organ. N Engl J Med (2003) 349(10):931–40. 10.1056/NEJMoa021744 12954741

[8] Bennett WM. Insights into chronic cyclosporine nephrotoxicity. Int J Clin Pharmacol Ther (1996) 34(11):515–9. 8937936

[9] Vincenti F Charpentier B Vanrenterghem Y Rostaing L Bresnahan B Darji P A phase III study of belatacept-based immunosuppression regimens versus cyclosporine in renal transplant recipients (BENEFIT study). Am J Transplant (2008) 10(3):535–46. 10.1111/j.1600-6143.2009.03005.x 20415897

[10] Vincenti F Larsen C Durrbach A Wekerle T Nashan B Blancho G Costimulation blockade with belatacept in renal transplantation. N Engl J Med (2005) 353(8):770–81. 10.1056/NEJMoa050085 16120857

[11] Ojo AO. Cardiovascular complications after renal transplantation and their prevention. Transplantation (2006) 82(5):603–11. 10.1097/01.tp.0000235527.81917.fe 16969281

[12] Nankivell BJ Borrows RJ Fung CL O’Connell PJ Allen RD Chapman JR. The natural history of chronic allograft nephropathy. N Engl J Med (2003) 349(24):2326–33. 10.1056/NEJMoa020009 14668458

[13] Glotz D Charpentier B Abramovicz D Lang P Rostaing L Rifle G Thymoglobulin induction and sirolimus versus tacrolimus in kidney transplant recipients receiving mycophenolate mofetil and steroids. Transplantation (2010) 89(12):1511–7. 10.1097/TP.0b013e3181db09e4 20386144

[14] Jacquet A Francois H Frangie C Ahmad L Charpentier B Durrbach A. Prevention of calcineurin inhibitor nephrotoxicity in renal transplantation. Transpl Immunol (2008) 20(1–2):29–31. 10.1016/j.trim.2008.09.002 18817870

[15] Büchler M Caillard S Barbier S Thervet E Toupance O Mazouz H Sirolimus versus cyclosporine in kidney recipients receiving thymoglobulin, mycophenolate mofetil and a 6-month course of steroids. Am J Transplant (2007) 7(11):2522–31. 10.1111/j.1600-6143.2007.01976.x 17868057

[16] Vincenti F Rostaing L Grinyo J Rice K Steinberg S Gaite L Belatacept and long-term outcomes in kidney transplantation. N Engl J Med (2016) 374(4):333–43. 10.1056/NEJMoa1506027 26816011

[17] Kremer JM Westhovens R Leon M Di Giorgio E Alten R Steinfeld S Treatment of rheumatoid arthritis by selective inhibition of T-cell activation with fusion protein CTLA4Ig. N Engl J Med (2003) 349(20):1907–15. 10.1056/NEJMoa035075 14614165

[18] Abrams JR Kelley SL Hayes E Kikuchi T Brown MJ Kang S Blockade of T lymphocyte costimulation with cytotoxic T lymphocyte-associated antigen 4-immunoglobulin (CTLA4Ig) reverses the cellular pathology of psoriatic plaques, including the activation of keratinocytes, dendritic cells, and endothelial cells. J Exp Med (2000) 192(5):681–94. 10.1084/jem.192.5.681 10974034 PMC2193278

[19] Levisetti MG Padrid PA Szot GL Mittal N Meehan SM Wardrip CL Immunosuppressive effects of human CTLA4Ig in a non-human primate model of allogeneic pancreatic islet transplantation. J Immunol (1997) 159(11):5187–91. 9548454

[20] Larsen CP Pearson TC Adams AB Tso P Shirasugi N Strobert E Rational development of LEA29Y (belatacept), a high-affinity variant of CTLA4-Ig with potent immunosuppressive properties. Am J Transplant (2005) 5(3):443–53. 10.1111/j.1600-6143.2005.00749.x 15707398

[21] Kirk AD Tadaki DK Celniker A Batty DS Berning JD Colonna JO Induction therapy with monoclonal antibodies specific for CD80 and CD86 delays the onset of acute renal allograft rejection in non-human primates. Transplantation (2001) 72(3):377–84. 10.1097/00007890-200108150-00005 11502964

[22] Montgomery SP Xu H Tadaki DK Celniker A Burkly LC Berning JD Combination induction therapy with monoclonal antibodies specific for CD80, CD86, and CD154 in nonhuman primate renal transplantation. Transplantation (2002) 74(10):1365–9. 10.1097/00007890-200211270-00002 12451232

[23] Hernández D Rufino M González-Posada JM Estupiñán S Pérez G Marrero-Miranda D Predicting delayed graft function and mortality in kidney transplantation. Transplant Rev (Orlando) (2008) 22(1):21–6. 10.1016/j.trre.2007.09.007 18631855

[24] Naesens M Kuypers DR Sarwal M. Calcineurin inhibitor nephrotoxicity. Clin J Am Soc Nephrol (2009) 4(2):481–508. 10.2215/CJN.04800908 19218475

[25] Durrbach A Pestana JM Pearson T Vincenti F Garcia VD Campistol J A phase III study of belatacept versus cyclosporine in kidney transplants from extended criteria donors (BENEFIT-EXT study). Am J Transplant (2010) 10(3):547–57. 10.1111/j.1600-6143.2010.03016.x 20415898

[26] Durrbach A Pestana JM Florman S Del Carmen Rial M Rostaing L Kuypers D Long-term outcomes in belatacept- versus cyclosporine-treated recipients of extended criteria donor kidneys: final results from BENEFIT-EXT, a phase III randomized study. Am J Transplant (2016) 16(11):3192–201. 10.1111/ajt.13830 27130868 PMC5516151

[27] Ferguson R Grinyó J Vincenti F Kaufman DB Woodle ES Marder BA Immunosuppression with belatacept-based, corticosteroid-avoiding regimens in de novo kidney transplant recipients. Am J Transplant (2011) 11(1):66–76. 10.1111/j.1600-6143.2010.03338.x 21114656

[28] Masson P Henderson L Chapman JR Craig JC Webster AC. Belatacept for kidney transplant recipients. Cochrane Database Syst Rev (2014) (11):CD010699. 10.1002/14651858.CD010699.pub2 25416857 PMC6486198

[29] Hart A Smith JM Skeans MA Gustafson SK Stewart DE Cherikh WS OPTN/SRTR 2015 annual data report: kidney. Am J Transplant (2017) 17(Suppl 1):21–116. 10.1111/ajt.14124 28052609 PMC5527691

[30] Rostaing L Massari P Garcia VD Mancilla-Urrea E Nainan G del Carmen Rial M Switching from calcineurin inhibitor-based regimens to a belatacept-based regimen in renal transplant recipients: a randomized phase II study. Clin J Am Soc Nephrol (2011) 6(2):430–9. 10.2215/CJN.05840710 21051752 PMC3052236

[31] Grinyo J Alberu J Contieri FL Manfro RC Mondragon G Nainan G Improvement in renal function in kidney transplant recipients switched from cyclosporine or tacrolimus to belatacept: 2-year results from the long-term extension of a phase II study. Transpl Int (2012) 25(10):1059–64. 10.1111/j.1432-2277.2012.01535.x 22816557

[32] Grinyó JM Del Carmen Rial M Alberu J Steinberg SM Manfro RC Nainan G Safety and efficacy outcomes 3 years after switching to belatacept from a calcineurin inhibitor in kidney transplant recipients: results from a phase 2 randomized trial. Am J Kidney Dis (2017) 69(5):587–94. 10.1053/j.ajkd.2016.09.021 27889299

[33] Le Meur Y Aulagnon F Bertrand D Heng AE Lavaud S Caillard S Effect of an early switch to belatacept among CNI-intolerant graft recipients of kidneys from extended criteria donors. Am J Transplant (2015) 16(7):2181–6. 10.1111/ajt.13698 26718625

[34] Brakemeier S Kannenkeril D Dürr M Braun T Bachmann F Schmidt D Experience with belatacept rescue therapy in kidney transplant recipients. Transpl Int (2016) 29(11):1184–95. 10.1111/tri.12822 27514317

[35] Gupta G Regmi A Kumar D Posner S Posner MP Sharma A Safe conversion from tacrolimus to belatacept in high immunologic risk kidney transplant recipients with allograft dysfunction. Am J Transplant (2015) 15(10):2726–31. 10.1111/ajt.13322 25988397

[36] Reynolds JC Agodoa LY Yuan CM Abbott KC. Thrombotic microangiopathy after renal transplantation in the United States. Am J Kidney Dis (2003) 42(5):1058–68. 10.1016/j.ajkd.2003.07.008 14582050

[37] Bren A Pajek J Grego K Buturovic J Ponikvar R Lindic J Follow-up of kidney graft recipients with cyclosporine-associated hemolytic-uremic syndrome and thrombotic microangiopathy. Transplant Proc (2005) 37(4):1889–91. 10.1016/j.transproceed.2005.02.112 15919494

[38] Lin CC King KL Chao YW Yang AH Chang CF Yang WC. Tacrolimus-associated hemolytic uremic syndrome: a case analysis. J Nephrol (2003) 16(4):580–5. 14696762

[39] Zarifian A Meleg-Smith S O’donovan R Tesi RJ Batuman V. Cyclosporine-associated thrombotic microangiopathy in renal allografts. Kidney Int (1999) 55(6):2457–66. 10.1046/j.1523-1755.1999.00492.x 10354295

[40] Sartelet H Toupance O Lorenzato M Fadel F Noel LH Lagonotte E Sirolimus-induced thrombotic microangiopathy is associated with decreased expression of vascular endothelial growth factor in kidneys. Am J Transplant (2005) 5(10):2441–7. 10.1111/j.1600-6143.2005.01047.x 16162193

[41] Karthikeyan V Parasuraman R Shah V Vera E Venkat KK. Outcome of plasma exchange therapy in thrombotic microangiopathy after renal transplantation. Am J Transplant (2003) 3(10):1289–94. 10.1046/j.1600-6143.2003.00222.x 14510703

[42] Cicora F Paz M Mos F Roberti J. Use of belatacept as alternative immunosuppression in three renal transplant patients with de novo drug-induced thrombotic microangiopathy. Case Rep Med (2013) 2013:260254. 10.1155/2013/260254 24198835 PMC3806345

[43] Ashman N Chapagain A Dobbie H Raftery MJ Sheaff MT Yaqoob MM. Belatacept as maintenance immunosuppression for postrenal transplant de novo drug-induced thrombotic microangiopathy. Am J Transplant (2009) 9(2):424–7. 10.1111/j.1600-6143.2008.02482.x 19120084

[44] Midtvedt K Bitter J Dorje C Bjorneklett R Holdaas H Belatacept as immunosuppression in patient with recurrence of hemolytic uremic syndrome after renal transplantation. Transplantation (2009) 87(12):1901–3. 10.1097/TP.0b013e3181a991ca 19543075

[45] Klintmalm GB Feng S Lake JR Vargas HE Wekerle T Agnes S Belatacept-based immunosuppression in de novo liver transplant recipients: 1-year experience from a phase II randomized study. Am J Transplant (2014) 14(8):1817–27. 10.1111/ajt.12810 25041339 PMC4140547

[46] Knechtle SJ Adams AB. Belatacept: is there BENEFIT for liver transplantation too? Am J Transplant (2014) 14(8):1717–8. 10.1111/ajt.12806 25041120

[47] LaMattina JC Jason MP Hanish SI Ottmann SE Klassen DK Potosky D Safety of belatacept bridging immunosuppression in hepatitis C-positive liver transplant recipients with renal dysfunction. Transplantation (2014) 97(2):133–7. 10.1097/01.TP.0000438635.44461.2e 24342980

[48] Timofte I Terrin M Barr E Sanchez P Kim J Reed R Belatacept for renal rescue in lung transplant patients. Transpl Int (2016) 29(4):453–63. 10.1111/tri.12731 26678245

[49] Enderby CY Habib P Patel PC Yip DS Orum S Hosenpud JD Belatacept maintenance in a heart transplant recipient. Transplantation (2014) 98(7):e74–5. 10.1097/TP.0000000000000404 25285956

[50] Martin ST Powell JT Patel M Tsapepas D. Risk of posttransplant lymphoproliferative disorder associated with use of belatacept. Am J Health Syst Pharm (2013) 70(22):1977–83. 10.2146/ajhp120770 24173007

[51] Grinyó J Charpentier B Pestana JM Vanrenterghem Y Vincenti F Reyes-Acevedo R An integrated safety profile analysis of belatacept in kidney transplant recipients. Transplantation (2010) 90(12):1521–7. 10.1097/TP.0b013e3182007b95 21088650

[52] Gupta G Abu Jawdeh BG Racusen LC Bhasin B Arend LJ Trollinger B Late antibody-mediated rejection in renal allografts: outcome after conventional and novel therapies. Transplantation (2014) 97(12):1240–6. 10.1097/01.TP.0000442503.85766.91 24937198

[53] Wiebe C Gibson IW Blydt-Hansen TD Karpinski M Ho J Storsley LJ Evolution and clinical pathologic correlations of de novo donor-specific HLA antibody post kidney transplant. Am J Transplant (2012) 12(5):1157–67. 10.1111/j.1600-6143.2012.04013.x 22429309

[54] Hidalgo LG Campbell PM Sis B Einecke G Mengel M Chang J De novo donor-specific antibody at the time of kidney transplant biopsy associates with microvascular pathology and late graft failure. Am J Transplant (2009) 9(11):2532–41. 10.1111/j.1600-6143.2009.02800.x 19843031

[55] Legendre C Canaud G Martinez F. Factors influencing long-term outcome after kidney transplantation. Transpl Int (2014) 27(1):19–27. 10.1111/tri.12217 24138291

[56] Wang CJ Heuts F Ovcinnikovs V Wardzinski L Bowers C Schmidt EM CTLA-4 controls follicular helper T-cell differentiation by regulating the strength of CD28 engagement. Proc Natl Acad Sci U S A (2015) 112(2):524–9. 10.1073/pnas.1414576112 25548162 PMC4299196

